# Population Genetic Structure of Apple Scab (*Venturia inaequalis* (Cooke) G. Winter) in Iran

**DOI:** 10.1371/journal.pone.0160737

**Published:** 2016-09-15

**Authors:** Leila Ebrahimi, Khalil-Berdi Fotuhifar, Mohammad Javan Nikkhah, Mohammad-Reza Naghavi, Niranjan Baisakh

**Affiliations:** 1 Department of Plant Pathology, Faculty of Agricultural Sciences and Engineering, College of Agriculture and Natural Resources, University of Tehran, Karaj, Iran; 2 Department of Biotechnology, Faculty of Agricultural Sciences and Engineering, College of Agriculture and Natural Resources, University of Tehran, Karaj, Iran; 3 School of Plant, Environmental and Soil Sciences, Louisiana State University Agricultural Center, Baton Rouge, LA, 70803, United States of America; National Bureau of Plant Genetic Resources, INDIA

## Abstract

The population genetic structure of 278 *Venturia inaequalis* isolates, collected from different apple cultivars of eighteen different provinces in Iran, was investigated using 22 polymorphic microsatellite markers. Analysis of molecular variation, Bayesian clustering and Nei's genetic distance analyses based on 88 microsatellite alleles indicated substantial levels of gene flow among the collection sites. Ninety three percent of the variation was observed among the individuals within the populations and only 7% variation was observed among the populations. Structure analysis grouped the isolates into two populations. Maximum number of pathogen genotypes (44) was observed in the North of Iran that grows various different apple cultivars. Investigation on the variation of the pathogen on different cultivars in the North of Iran suggested a significant differentiation of the pathogen populations between wild apple and commercial cultivars. During sampling, varying ranges of scab infection were observed on various apple cultivars in forests, monoculture and mix orchards. Wild type apple (*Malus orientalis*) along the Caspian Sea Coast had the most infection in comparison with the Iranian endemic and commercial cultivars. Based on the genetic analysis and host tracking scenario of the pathogen, it was presumed that Iran could potentially be the center of origin of *V*. *inaequalis*, which requires further detailed studies with isolates collected from different parts of central Asia and world for confirmation.

## Introduction

Scab caused by *Venturia inaequalis* (Cooke) G. Winter is one of the most important diseases of the apple growing regions worldwide [1], especially in regions with cool and wet spring and early summer [2]. It is considered as one of the most serious threats to commercial apple production [2] causing severe reduction in the quality and size of fruits, premature fruit drop, defoliation and reduction of tree vigor over time [3]. Apple scab occurrence was first recorded from Sweden in 1819 and Germany in 1833 [2]. It was first reported in Iran in 1946 [4].

*Venturia inaequalis* is a heterothallic Ascomycetes fungus that overwinters as pseudothecia in the leaf litter, whereas in regions with moderate winter it survives as conidia in dormant buds [5]. The life cycle of the pathogen is comprised of one sexual and multiple asexual reproductions annually, which causes significant variations in the fungus population. Annual sexual reproduction leads to recombination and high variation in fungal genome and changes in population genetic structure. Variation in the pathogen population is one the most important factors for consideration in devising the management strategies of the disease. Rapid evolution of new races of fungi that overcome the resistance genes in the host and also fungicides that leads to problems in the control of the disease.

*Venturia inaequalis* is known to overcome host resistance genes [6]. The ability of the pathogen populations to resist fungicide and the dearth of resistant cultivars with desirable agronomic traits are the increasing challenges of apple scab management [7]. Development of resistant cultivars is the most effective, economically sustainable and environmentally friendly method of disease control. Apple has some cultivars that possess resistance genes to scab, but some genes, such as *Rvi6* (*Vf*), have been overcome by the pathogen in several regions [8]. Bus et al. [6], from a study on identifying differential *Malus* hosts carrying single resistance genes for avirulence genes of *V*. *inaequalis*, provided the first hand information on the 17 gene-for-gene relationships. Based on the information of identifying the complex races of *V*. *inaequalis*, a long-term method of plant breeding could provide with resistant cultivars can be provided with the resistance sources carrying pyramided resistances [6]. To achieve this goal, detail investigations on the variation and population genetic structure of the pathogen in different regions is required.

Genetic variation and population structure of *V*. *inaequalis* were studied in Czech Republic [9], Spain [10], Sweden [11], Brazil [12], India [13], and Pennsylvania [7] and Minnesota [14] within the USA. A comparison of population structure between Asian (China and India) and UK isolates showed more pathogen diversity in the European population [15]. Population genetic structure analysis of *V*. *inaequalis* collected from around the world showed that genetic diversity within the populations was more than that among the populations [16]. Based on this study, the Central Asia has been known as the origin of this economical disease. This scenario was approved based on the variability among the pathogen populations, along with coalescent analyses of migration models and estimates of genetic distances.

Population genetic structure of a pathogen reflects its history and evolutionary potential [17]. Similarly, genetic diversity can provide clues on the centers of origin of the pathogen where it has the greatest diversity [18]. Host-tracking scenario suggests the coevolution of the host and the pathogen during the process of host domestication and development of the agro-ecosystem specific to the host crop. So the origin of the pathogen is expected to be the same with the host origin [18].

Apple is the most common and culturally important fruit crop worldwide. The center of origin of apple is considered to be the mountain ranges of Central Asia along the Silk Roads stretching from Asia to Europe [19]. Based on the evidences and information to date, apple cultivation possibly began in the region between the Caspian and Black seas, which subsequently reached the Near East nearly 3000 years ago [20]. Gharghani et al. [21], with an objective to determine the role of Iran in apple evolution and domestication, investigated the relationships of wild and domesticated apples in the world; however, they did not survey the Iranian wild apple during their study. Based on their results, Iranian apples seem to be the intermediates between the domesticated varieties and wild species. So, Iran was assumed to be one of the major players in the domestication and transfer of apples from Central Asia to the Western countries. Keeping in view the origin of apple and *V*. *inaequalis*, Iran can be considered as an important region in the distribution of *V*. *inaequalis* along the Silk Road. Apple is one of the superior crops in Iran due to its nutritional and export value. According to the Food and Agriculture Organization (FAO, 2012), Iran is ranked as the seventh largest country in the world for apple production. Apple scab is endemic to Iran because of the suitable environmental conditions in apple orchards for *V*. *inaequalis* (cold and wet weather in spring and early summer); it is one of the serious threats for agricultural economy in Iran. However, comprehensive information on the genetic structure of apple scab disease in Iran is lacking. The present study was undertaken with an objective of investigating the variation of this pathogen on different apple cultivars in different regions of Iran, and reports on the population genetic structure of *V*. *inaequalis* in Iran.

## Materials and Methods

### Collection of infected apple samples

**Fig 1:**
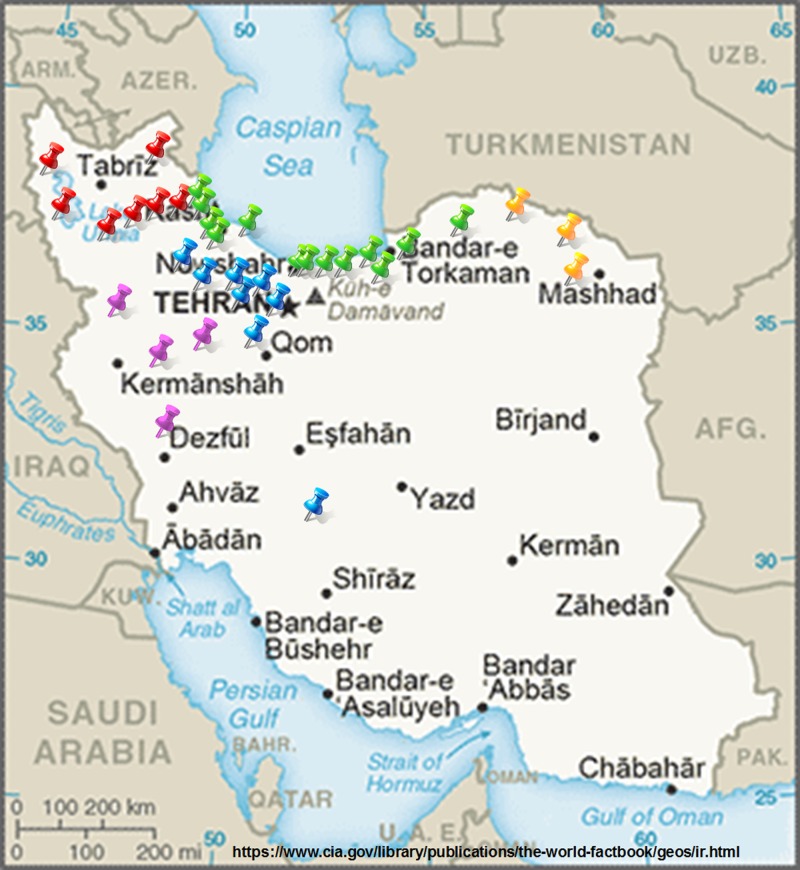
Geographic locations of *Venturia inaequalis* sampling sites in Iran. Different populations are represented by different color filled circles: Northwest population (red); West (purple); Central (blue); North (green), and Northeast (yellow).

Five hundred and forty infected samples were collected from 18 provinces of Iran (Alborz, Ardabil, Eastern Azarbaijan, Golestan, Guilan, Hamadan, Isfahan, Kermanshah, Kurdistan, Lorestan, Markazi, Mazandaran, North Khorasan, Qazvin, Razavi Khorasan, Tehran, Western Azarbaijan, and Zanjan) during years 2013 and 2014 (S1 Table). The owner of the land on each site gave permission to collect the samples from their field. Further, the field studies did not involve endangered or protected species. The geographic locations of the sampling sites are provided in Fig 1. Samples including apple leaves and fruits with scab symptoms, and litter leaves with mature ascospores were collected during May to October, and March to early May, respectively. Sampling was done randomly on different apple cultivars including wild apple (*Malus orientalis*), Iranian endemic cultivars (*Malus domestica*: “Abbasi”, “Bahareh”, “Golab”, “Ghandak”, “Moruei”, “Rasmi”, “Sibe sang”, “Sorkh”, “Shafiabadi”) and commercial cultivars (*Malus domestica*: “Golden delicious” and “Red delicious”). Sampling was done from the trees where there was at least one spot on a leaf.

The fungus from the infected samples was isolated using single spore method by streaking out spores on plates containing 2% water agar (WA) and culturing of single germinated conidium on potato dextrose agar (PDA). Pure fungal cultures were obtained by transferring single germinated spore on PDA. Infected leaves were also collected in 2009 and maintained at the arboretum of the University of Tehran, Karaj. Fungal spores were isolated from these leaves for further studies.

### DNA extraction and genotyping

Fungal isolates were cultured on cellophane discs placed on PDA for 10 to 14 days at 16–18°C under continuous dark. Mycelia were collected and after freeze-dried, subjected to DNA extraction using Iraizol DNA extraction buffer (RNA Biotechnology Co., Iran). DNA extraction of a few isolates was conducted directly from infected leaf showing symptoms according to Iraizol DNA extraction protocol. DNA was quantified and quality-checked using a ND-1000 spectrophotometer (Nanodrop Technologies, Wilmington, DE) and was diluted to a working concentration of 25 ng/μl and stored at -20°C until further use.

Twenty eight published SSR primer pairs [22, 23] were used to genotype the fungal populations. PCR was performed in a 10 μl final volume containing 2 μl of 5X PCR buffer, 3 mM MgCl_2_, 0.16 mM dNTP, 0.2 μl of *Taq* DNA polymerase (Promega, Madison, WI), 2 μM of each primer and 2 μl DNA template. PCR amplification was carried out in thermal Cycler (Bio-rad, Hercules, CA) using the following conditions: an initial denaturation at 94°C for 5 min, followed by 30 cycles of denaturation step at 94°C for 40 s, 40 s of annealing at 55°C, 40 s of extension at 72°C, and a final extension of 10 min at 72°C. The amplified PCR products were resolved in 3.5% Agarose SFR (Amresco, Solon, OH) gel at 100 V for 4 h and visualized using a Kodak Gel Logic 200 documentation system (Kodak Inc, New Brokhaven, CT). Allele sizes were determined using the Molecular Image Analyzer software (Carestream Health Inc, Rochester, NY).

### Genetic differentiation among *V*. *inaequalis* isolates on different apple cultivars in the North of Iran

The North of Iran (including Golestan, Guilan and Mazandaran provinces) has more number of different apple cultivars in comparison with other regions of Iran. During sampling, wild apple was found only in the North. Hence, 51 isolates of different apple cultivars from North population were selected to investigate their genetic diversity using polymorphic microsatellite markers.

### Statistical analysis

The PCR-generated bands were scored as ‘1’ (for presence) and ‘0’ (absence) in a binary matrix for further analysis. To check whether the loci are neutral or targeted by natural selection, Ewans-Waterson test was simulated with PopGene ver. 1.32 [24]. Nei's genetic distance among the populations were estimated and the proportion of shared alleles was calculated using PopGene and GenALex ver. 6.5 [25]. The average gene diversity [26] and the average number of alleles per locus were estimated from the datasets using GenALex and Arlequin ver. 3.5 [27], respectively. Private alleles were estimated from GenALex analysis. Population differentiation and gene flow were estimated by F_ST_ and N_m_, respectively, using GenALex. The genetic diversity within a population (H_s_) and total heterozygosity (H_t_) for every locus, and expected heterozygosity (H_e_) and observed heterozygosity (H_o_) for every population calculated with PopGene and Arlequin.

A hierarchical analysis of molecular variance (AMOVA) was performed using the GenALex and Arlequin using default parameters to identify the distribution of population substructure at different geographic scales. The average number of alleles per locus was estimated using Arlequin. An unweighted pair group method with arithmetic mean (UPGMA) tree was produced with Nei's [26] Genetic distance using PopGene.

Assignment of individuals to a specified number of clusters (K) and population ancestry was done using Structure ver. 2.3.4 software [28], which implements a clustering algorithm based on a Bayesian model. Assuming random mating there should be one population (i.e. K = 1); if there is sufficient population differentiation, K is expected to be greater than one. To estimate the number of clusters, an admixture model with correlated allele frequencies was run 10 times, with 10000 iterations followed by 100000 Markov chain Monte Carlo interactions for K = 1 to 10. ΔK method [29] was used to best estimate K, which was computed using Structure Harvester ver. 0.56.3 [30]. The distribution of the highest value of the ancestry coefficient for each K was analyzed following Frantz et al. [31]. Individuals were assigned to a single cluster when the proportion of ancestry in the cluster was greater than 80%. Based on this threshold, the assignment rate for each K was computed as the proportion of individuals assigned to a single cluster (i.e. with a proportion of ancestry over the 80% threshold).

Associations of alleles among different loci were examined in each isolate using the index of association *r*_*d*_ statistics [32, 33], which is a generalized measure of multilocus linkage disequilibrium [34]. The null hypothesis of random association of alleles, consistent with random mating, was tested using the Multilocus software [33] by comparing the observed value of the statistic to that obtained after 1000 randomizations to simulate recombination and Arlequin.

## Results

**Fig 2:**
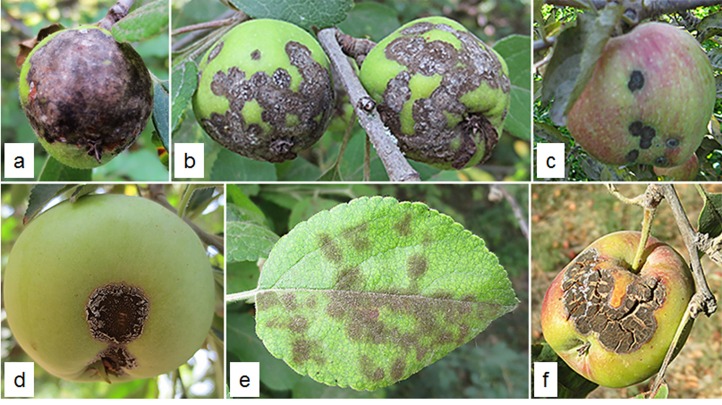
Ranges of apple scab symptoms on different apple cultivars in Iran. a-b, Wild apple (*Malus orientalis*); c, Red delicious (*Malus domestica*); d-f, Iranian endemic cultivars (*Malus domestica*). Photographs were taken during July and August, 2014 when the fruits and leaves were 2-4-months-old depending on the varieties.

During sampling, a range of differential scab infections was observed on different cultivars in forests, monoculture and mixed orchards (Fig 2). All of the wild apple trees in the forests along the Caspian Sea Coast had strong infection symptoms on the leaves and fruits (Fig 2A and 2B). Red delicious had the most scab infection among all the commercial cultivars (Fig 2C), whereas Golden delicious was rarely infected in some mixed infected orchards. In mixed infected orchards, most of the Iranian endemic cultivars had high level of infection on leaves and fruits (Fig 2D–2F). But, there were some Iranian endemic cultivars that were not infected with scab.

Of the 540 scab-infected samples, 280 isolates from different regions were selected for investigation of their population genetic structure. Genomic DNA of two isolates could not be amplified with the SSR primers tested, so these two isolates were not included in further genetic analysis. The remaining 278 isolates were divided into five populations based on their geographical origin, such as Northwest, West, Central, North and Northeast (Table 1). Of the 28 SSR markers screened for population genotyping, five (Vitc1/82, viga3/z, viaacs10, vigt8/146, 1aac4h) were monomorphic for all individuals within and among populations, and one marker (vitcca7/p) did not have amplification product in 13 individuals. Thus, these six markers were excluded and the alleles from 22 markers were used for genotypic analysis.

**Table 1:** Genetic diversities in different populations of *Venturia inaequalis*.

Population	N	N_a_	N_e_	P_a_	k	h	uh	I	H_e_	H_o_
Northwest	61	77	1.30	2	42	0.19	0.20	0.30	0.40	0.02
West	50	65	1.27	-	34	0.17	0.17	0.27	0.43	0.02
Central	54	69	1.28	-	33	0.18	0.18	0.28	0.44	0.01
North	62	71	1.29	1	44	0.18	0.18	0.30	0.40	0.03
Northeast	51	76	1.28	3	40	0.18	0.19	0.30	0.39	0.03

N, sample size; N_a_, number of alleles; N_e_, number of effective alleles; P_a_, private alleles; k, number of genotypes; h, gene diversity (Nei); uh, unbiased diversity; I, shannon index; H_e_, expected heterozygosity; H_o_, observed heterozygosity.

Genotype of each isolate was defined as the combination of alleles for the 22 SSR loci tested. Among the 278 isolates analyzed, 33 to 44 genotypes in different populations were observed based on a total of 88 different alleles. The number of effective alleles in every population was 1.27 to 1.3 (Table 1) and the number of alleles at each locus ranged from two (Vigt10/ɛ and Viaggt8/1) to 11 (1tc1g), with an average value of 4 (Table 2). Overall, six private population-specific alleles were identified—two unique alleles in Northwest population, one in North and three in Northeast population.

**Table 2:** Summary of the genetic analysis of *Venturia inaequalis* isolates with 22 SSR loci.

Locus	Allele Size (bp)	N	N_a_	N_e_	h	I	H_e_	H_o_	H_s_	H_t_	F_st_	F_st_-P-value
Vitc1/2	170–290	4.6	6	1.38	0.23	0.35	0.67	0.68	0.22	0.23	0.048	0.26
Vitc2/D	170–210	3.8	5	1.48	0.27	0.41	0.66	0.66	0.27	0.27	0.015	0.02
vitc2/16	150–170	2.0	4	1.06	0.05	0.12	0.10	0.10	0.05	0.05	0.080	0.25
vica9/x	195–210	2.8	3	1.33	0.24	0.39	0.33	0.34	0.22	0.24	0.070	0.39
Vigt10/ɛ	171–173	1.8	2	1.03	0.03	0.09	0.03	0.03	0.04	0.04	0.002	0.04
vitg9/99	180–190	2.4	3	1.07	0.07	0.15	0.10	0.10	0.20	0.07	0.010	0.10
vitg9/129	280–290	2.8	3	1.24	0.18	0.33	0.28	0.28	0.18	0.19	0.030	0.19
vica9/134	210–230	3.0	3	1.48	0.30	0.47	0.45	0.46	0.28	0.30	0.090	0.25
Vitg11/70	190–200	3.0	3	1.53	0.31	0.48	0.47	0.49	0.26	0.30	0.170	0.02
Vict1/130	145–160	2.4	3	1.08	0.07	0.16	0.11	0.11	0.07	0.07	0.030	0.27
Viaggt8/1	195–199	2.8	3	1.02	0.02	0.06	0.02	0.20	0.02	0.19	0.040	0.06
Viga7/116	140–180	4.6	5	1.40	0.24	0.41	0.60	0.62	0.27	0.26	0.150	0.04
Vica9/152	180–195	1.2	2	1.37	0.30	0.49	0.48	0.49	0.27	0.30	0.110	0.15
Vigtg10/95	150–170	3.4	4	1.51	0.28	0.41	0.55	0.56	0.26	0.28	0.050	0.37
vica10/154	110–195	5.6	7	1.37	0.22	0.34	0.72	0.73	0.20	0.22	0.060	0.43
Vicacg8/42	195–240	3.2	4	1.47	0.28	0.42	0.56	0.57	0.25	0.28	0.100	0.18
1tc1a	105–180	5.4	6	1.39	0.24	0.40	0.77	0.77	0.25	0.25	0.030	0.06
1tc1b	150–190	2.6	3	1.29	0.21	0.36	0.32	0.32	0.20	0.20	0.040	0.29
1tc1g	110–200	7.6	11	1.24	0.17	0.28	0.82	0.83	0.15	0.17	0.600	0.34
1aac4b	180–190	2.2	3	1.06	0.05	0.13	0.08	0.08	0.05	0.06	0.040	0.40
1aac4f	110–125	2.0	3	1.04	0.04	0.10	0.06	0.06	0.04	0.04	0.180	0.19
1aac3b	125–130	2.0	2	1.21	0.18	0.32	0.18	0.18	0.16	0.17	0.080	0.30

N, average number of alleles; N_a_, total number of alleles; N_e_, number of effective alleles; h, gene diversity (Nei); I, shannon index; H_e_, expected heterozygosity; H_o_, observed heterozygosity; H_s_, genetic diversity within the population; H_t_, total heterozygosity; F_st_, genetic differentiation.

Gene diversity was comparable among the populations, ranging from 0.17 to 0.19 (Table 1). Shannon-Wiener′s index (I),) an estimate of diversity, for the five populations ranged from 0.27 to 0.30, indicating an overall average diversity of *V*. *inaequalis* within the populations (Table 1). Based on r_d_ (0.0053, P-value = 0.021), random mating was evident among individuals and Hardy-Weinberg equilibrium was apparent in all five populations. The observed heterozygosity within individuals for all populations was comparable but significantly lower than the expected heterozygosity.

A hierarchical AMOVA revealed the distribution of population substructure at different geographic scales. While most of the variation (93%) was explained among the individuals within a population, a significant proportion of the variation (7%) was also attributable to differences among populations from different regions (Table 3).

**Table 3:** Hierarchical analysis of molecular variance of *Venturia inaequalis* in five populations from different regions of Iran, and three populations on different cultivars in the North of Iran.

Source of variation	Degrees of freedom	Sum of squares	Percentage variation
Among population from different regions	4	148.94	7
Within population	551	2166.51	93
Total	555	2315.45	100
Among population from different cultivars	2	23.02	3
Within population	48	353.14	97
Total	50	376.16	100

All pairwise population differences were statistically significant (P = 0.001) (Table 4). The F_ST_ values showed an average value of differentiation between different populations and that the five populations had different proportion of migration and gene flow between each other. The total value of F_ST_ was 0.07 (P-value = 0.001) with the theta value of 0.075 (P-value = 0.021). The F_ST_ values were consistently highest between North and West populations with a minimum gene flow (N_m_ = 4.88). The gene flow was highest between West and Central populations (N_m_ = 26.09).

**Table 4:** Pairwise F_ST_ (diagonal vales; P = 0.001) and N_m_ calculated with GeneALex for *Venturia inaequalis* populations in Iran.

Population	North West	West	Central	North	North East
North West		8.18	9.69	7.69	6.41
West	0.058		26.09	4.88	5.91
Central	0.05	0.019(P = 0.003)		5.36	5.79
North	0.061	0.093	0.085		7.67
North East	0.072	0.078	0.079	0.061	

**Fig 3:**
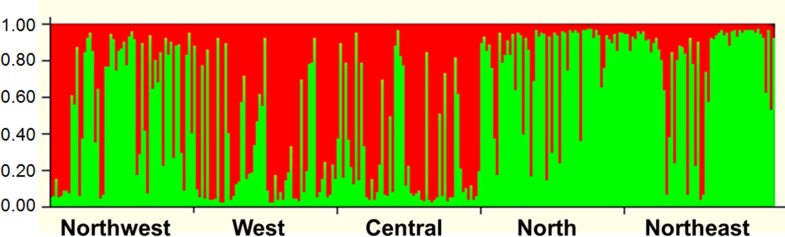
Population structure showing ancestry coefficient of *Venturia inaequalis* in Iran using the Structure bar plot (K = 2).

**Fig 4:**
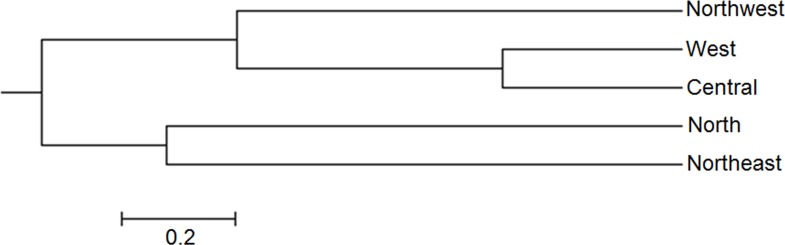
Unweighted pair group with arithmetic mean (UPGMA) dendrogram based on Nei's genetic distance between five *Venturia inaequalis* populations with 22 SSR markers.

Structure analysis revealed that isolates of *V*. *inaequalis* collected from different places of Iran were divided into two populations (K = 2) (Fig 3). Isolates from Northwest, North and Northeast that are in the same latitude in Iran geographical map were grouped as one population. Similarly, isolates from Central and West areas that are in the same geographical latitude composed one population (Fig 3). Moreover, STRUCTURE analysis grouped 64 individuals (23% of the total isolates) with a Q admixture proportion to the first cluster with the probability of 0.2 and 0.8, suggesting a substantial level of gene flow between the two clusters. The dendrogram based on Nei's [26] genetic distance using UPGMA of the isolates from five different geographic populations also showed that isolates were grouped into two major clusters (Fig 4).

### Genetic differentiation between *Venturia inaequalis* isolates of different apple cultivars in the North of Iran

**Fig 5:**
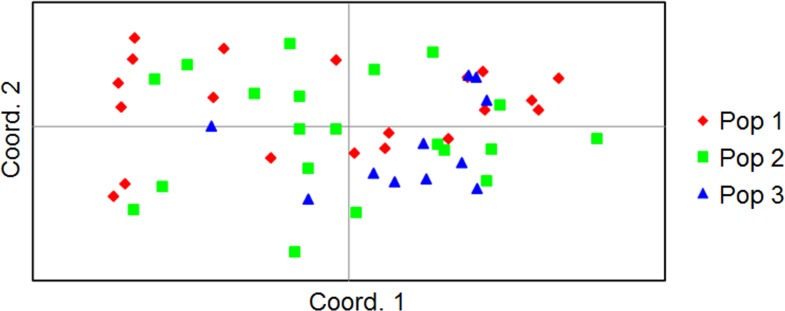
Principal coordinate analysis showing the clustering of three *Venturia inaequalis* populations from different apple cultivars in the North of Iran. Coord. 1, coordinate 1 (11.4%), Coor. 2, coordinate 2 (10.52). Pop 1, wild apple; Pop 2, Iranian endemic cultivars; Pop 3, commercial cultivars.

AMOVA results showed that 97% of the genetic variation was distributed among the individuals within the populations and only 3% of the variation was attributable to differences among the populations (Table 3). F_ST_ revealed a significant differentiation between wild and commercial apple cultivars. Also, based on F_ST_ and N_m_, Iranian endemic cultivars had apparently more gene flow with wild apple than commercial cultivars. Overall, Iranian endemic cultivars did not show significant differentiation with wild apple and commercial cultivars (Table 5). This result was also validated by the principal coordinate analysis (PCoA), where coordinate 1 and 2 explained for 11.4 and 10.52% of the variations (Fig 5).

**Table 5:** Pairwise F_ST_ (diagonal values) and N_m_ calculated with GeneALex for *Venturia inaequalis* populations on different apple cultivars in the North of Iran.

Population	Wild apple	Iranian endemic cultivars	Commercial cultivars
Wild apple		31.177	5.412
Iranian endemic cultivars	0.016 (P = 0.15)		30.626
Commercial cultivars	0.085 (P = 0.003)	0.016 (P = 0.175)	

## Discussion

Genotyping of the *V*. *inaequalis* populations of Iran by 22 microsatellite markers showed high genetic variation within the populations (93%), while there was low variation among the populations (7%) (Table 3). Similar results were obtained in previous studies of genetic variation of *V*. *inaequalis* in Minnesota [14] and in different countries from five continents [16]. High recombination in sexual reproduction and increase in these isolates via asexual reproduction during the spring and summer may result in this level of variation within and between the populations, respectively, of the heterothallic and hemi-biotroph fungus *V*. *inaequalis*. Based on r_d_, random mating was apparent among the isolates and Hardy-Weinberg equilibrium was observed in all five populations. Sexual reproduction is one of the most important factors in maintaining diversity within the populations as well as the survival of the fungus. Gene flow via transfer of the asexual propagules between the populations established in different geographic locations could be another factor that affects the variation [35, 36].

Maximum number of pathogen genotypes (44) was observed in the North of Iran (Table 1) where there is more number of different types of apple cultivars. Increased diversity of a cultivated plant in a certain region may result from long and intense cultivation, ecological diversity, and/or introgression of wild crop relatives, and thus knowledge about the cultivation history of the crop is also needed [18]. During sampling, wild apple (*M*. *orientalis*) was found only in the forests of North of Iran with a severe infection with apple scab. Samples were also collected from different Iranian endemic apple cultivars in the North, but the commercial apple cultivars were rare in the North, or were uninfected with scab in the mixed infected orchards. So, the variation in fungal genotypes in the North in comparison with other populations can be because of the diversity of the apple cultivars and suitable weather condition of cold and wet early spring. Northwest Iran had the second highest number of genotypes (42). In Northwest of Iran, apple is cultivated as an important and valuable crop. The cold weather of this area is conducive for *V*. *inaequalis*. Orchards in West Azerbaijan were severely infected with scab, especially on Red delicious. Golden delicious was infected less than other cultivars in all regions.

F_ST_ values between different populations showed highest genetic differentiation between North and West populations (F_ST_ = 0.093; N_m_ = 4.88). This could be because of the long physical distance and geographical barriers byt Alborz and Zagros Mountains that restrict pathogen migration between the two regions. Maximum migration and gene flow was observed between West and Central populations that resulted in less differentiation of the isolates between these two regions (Table 4). This was also evident from the UPGMA dendrogram based on Nei's genetic distance where West and Central populations were in the same clade with low genetic distance (Fig 4). Transmission of propagules via wind and transfer of infected plant materials between regions is the casual factor of gene flow. Gene flow results in isolates with different alleles in every population. Thus, pathogens with high gene flow rate between populations are able to overcome the host resistance and become resistant to fungicides. So, pathogens like *V*. *inaequalis* with a mixed reproduction system, has a high potential for gene flow via asexual propagules and high mutation rates that are serious threats to agriculture [36].

The present research investigated population genetic structure of *V*. *inaequalis* from different cultivars of different places of Iran. Information on the genetic variability in a pathogen population is important for determining appropriate disease management strategies, particularly for development of host resistance. Breeding for disease resistance may benefit from the genetic structure of a plant pathogen population that reflects its history and evolutionary potential [17]. In addition, genetic diversity is used to infer the centers of origin of the pathogen where the pathogen has greatest diversity [18]. The host and the pathogen are expected to coevolve during the process of host plant domestication and the development of crop specific agro-ecosystem specific. So the origin of pathogen is expected to be same as the host [18]. Structure and Structure Harvester analyses grouped the isolates into two populations (K = 2) (Fig 3). Isolates from Northwest, North and Northeast that are in the same latitude in Iran geographical map (Fig 1) formed one population. Conidia migration via wind between these regions could be one of the most important factors in establishing the genetic structure of the pathogen in these regions. Similarly, isolates from Central and West regions that are in the same geographical latitude (Fig 1) composed the other population. North of Iran is separated from the Central part by Alborz Mountains as the geographical barrier of pathogen migration via wind or other agents between the two regions. But, the presence of 23% admixture individuals suggested a substantial level of gene flow between the two clusters. More admixture individuals were present in Northwest population than other populations, which could be because of the migration between this population and other populations, especially West and Central population that are geographically less distant. Also, different apple varieties are cultivated mostly in Northwest of Iran that has a favorable environmental condition for apple as well as *V*. *inaequalis*. Seedlings with different resistance genes are derived from different sources (regions) in Iran, which increases the probability of the presence of different pathogen genotypes.

During collections of isolates from different domesticated and undomesticated apples from different regions of Iran, different infection range was observed on cultivars in forests, monoculture and mix orchards (Fig 2). Interestingly, wild apple trees had the most infection rate based on the proportion of infected trees where scab symptom was strong and widespread in the fruits and leaves along the Caspian Sea Coast. Iranian endemic cultivars had the least level of scab infection with the presence of some uninfected Iranian endemic cultivars in mixed infected orchards. The commercial cultivars had different infection rates that were lower than the wild apple. These observations provided clues that *V*. *inaequalis* had been in Iran before the domestication of apple, and thus, the pathogen was able to adapt and overcome the resistance genes in wild apples during its long pathogenic life [18].

Genetic analysis of the isolates collected from different cultivars in the North of Iran with 18 polymorphic SSR loci showed significant differentiation between wild apple and commercial cultivars populations, which suggested a low level of gene flow between these two populations. Iranian endemic cultivars had more gene flow with wild apple than commercial cultivars. But, Iranian endemic cultivars did not show significant differentiation from commercial cultivars. The present results on apple scab pathogen based host tracking are in agreement with the results of Gharghani et al. [21] that showed that Iranian apples may occupy an intermediate position between the domesticated varieties and wild apples. However, their research did not include a survey of the Iranian wild apple. The present and previous results suggest an important scenario about *V*. *inaequalis* evolution in Iran and also in the world, that this pathogen existed in Iran for a long time before apple cultivation and that Central Asia, especially Iran, is the probable center of origin of *V*. *inaequalis* in the world. However, further extensive studies including identification of resistance genes in different apple cultivars, analysis of the pathogen populations based on resistance genes, and comparison of other isolates from Central Asia and around the world would validate the present presumption on the origin of the pathogen.

## Supporting Information

S1 Table Detail information of the geographic location, apple cultivars, and the year that the isolates were collected and used for genotyping. (DOCX)
