# Correction: Population Genetic Structure of Apple Scab (*Venturia inaequalis* (Cooke) G. Winter) in Iran

**DOI:** 10.1371/journal.pone.0167415

**Published:** 2016-11-22

**Authors:** Leila Ebrahimi, Khalil-Berdi Fotouhifar, Mohammad Javan Nikkhah, Mohammad-Reza Naghavi, Niranjan Baisakh

The second author’s name is spelled incorrectly. The correct name is: Khalil-Berdi Fotouhifar. The correct citation is: Ebrahimi L, Fotouhifar K-B, Javan Nikkhah M, Naghavi M-R, Baisakh N (2016) Population Genetic Structure of Apple Scab (Venturia inaequalis (Cooke) G. Winter) in Iran. PLoS ONE 11(9): e0160737. doi:10.1371/journal.pone.0160737
