# Supplementary material for: Population Genetic Structure of Apple Scab (Venturia inaequalis (Cooke) G. Winter) in Iran
Source: PLoS One. 2016 Sep 15;11(9):e0160737. doi: 10.1371/journal.pone.0160737 (PMC5025049; doi:10.1371/journal.pone.0160737)
Supplement: S1 Table — (DOCX) [file pone.0160737.s001.docx]

S1 Table. Detail information of the geographic location, apple cultivars, and the year that the isolates were collected and used for genotyping

| **Year of Collection** |  | **Host Cultivar** | **Location** | **Provinve** | **Sample ID** | **N** |
| --- | --- | --- | --- | --- | --- | --- |
| Northwest population | | | | | | |
| 2013 | 37º62.928ʹN  47º78.163ʹE | Golab | Mianeh, Kandovan | East Azerbaijan | VI61 | 1 |
| 2013 | 37º62.934ʹN  47º78.153ʹE | Golden Delicious | Mianeh, Kandovan | East Azerbaijan | VI62 | 2 |
| 2013 | 34º47.35ʹN  48º28.621ʹE | Golab | Mianeh, Kandovan | East Azerbaijan | VI70 | 3 |
| 2013 | 34º47.359ʹN  48º28.7ʹE | Red Delicious | Mianeh, Kandovan | East Azerbaijan | VI73 | 4 |
| 2013 | 34º47.34ʹN  48º28.674ʹE | Red Delicious | Mianeh, Kandovan | East Azerbaijan | VI76 | 5 |
| 2013 | 34º47.3ʹN  48º28.811ʹE | Golden Delicious | Mianeh, Kandovan | East Azerbaijan | VI79 | 6 |
| 2013 | 37º70.915ʹN  47º76.411ʹE | Golden Delicious | Mianeh, Kandovan | East Azerbaijan | VI115 | 7 |
| 2013 | 37º70.922ʹN  47º76.763ʹE | Golden Delicious | Mianeh, Kandovan | East Azerbaijan | VI118 | 8 |
| 2013 | 37º71.076ʹN  47º76.325ʹE | Red Delicious | Mianeh, Kandovan | East Azerbaijan | VI125 | 9 |
| 2013 | 37º74.917ʹN  47º96.053ʹE | Red Delicious | Mianeh, Kandovan | East Azerbaijan | VI138 | 10 |
| 2013 | 37º74.982ʹN  47º95.933ʹE | Red Delicious | Mianeh, Kandovan | East Azerbaijan | VI140 | 11 |
| 2013 | 37º79.379ʹN  47º96.279ʹE | Red Delicious | Mianeh, Kandovan | East Azerbaijan | VI153 | 12 |
| 2013 | 37º79.407ʹN  47º96.283ʹE | Red Delicious | Mianeh, Kandovan | East Azerbaijan, | VI157 | 13 |
| 2014 | 37º75.114ʹN  47º95.066ʹE | Red Delicious | Mianeh, Kandovan | East Azerbaijan | VI288 | 14 |
| 2014 | 37º37.085ʹN  47º46.768ʹE | Red Delicious | Mianeh, Kandovan | East Azerbaijan | VI435 | 15 |
| 2014 | 37º37.08ʹN  47º46.769ʹE | Red Delicious | Mianeh, Kandovan | East Azerbaijan | VI437 | 16 |
| 2014 | 37º37.105ʹN  47º46.77ʹE | Red Delicious | Mianeh, Kandovan | East Azerbaijan | VI439 | 17 |
| 2013 | 37º52.868ʹN  47º35.296ʹE | Red Delicious | Mianeh, Torkamanchay | East Azerbaijan | VI166 | 18 |
| 2013 | 37º52.786ʹN  47º34.935ʹE | Red Delicious | Mianeh, Torkamanchay | East Azerbaijan | VI176 | 19 |
| 2013 | 37º53.106ʹN  47º34.635ʹE | Red Delicious | Mianeh, Torkamanchay | East Azerbaijan | VI178 | 20 |
| 2013 | 37º40.36ʹN  46º23.076ʹE | Red Delicious | Maragheh | East Azerbaijan | VI86 | 21 |
| 2013 | 37º40.579ʹN  46º23.188ʹE | Red Delicious | Maragheh | East Azerbaijan | VI89 | 22 |
| 2013 | 37º40.783ʹN  46º23.385ʹE | Red Delicious | Maragheh | East Azerbaijan | VI95 | 23 |
| 2013 | 37º40.306ʹN  46º23.059ʹE | Red Delicious | Maragheh | East Azerbaijan | VI99 | 24 |
| 2013 | 37º41.526ʹN  46º23.995ʹE | Red Delicious | Maragheh | East Azerbaijan | VI105 | 25 |
| 2013 | 37º41.26ʹN  46º24.312ʹE | Red Delicious | Maragheh | East Azerbaijan | VI107 | 26 |
| 2013 | 37º41.131ʹN  46º22.733ʹE | Red Delicious | Maragheh | East Azerbaijan | VI112 | 27 |
| 2013 | 38º17.777ʹN  44º71.8ʹE | Red Delicious | Salmas | West Azerbaijan | VI158 | 28 |
| 2013 | 38º17.183ʹN  44º73.482ʹE | Red Delicious | Salmas | West Azerbaijan | VI159 | 29 |
| 2013 | 38º17.521ʹN  44º77.48ʹE | Red Delicious | Salmas | West Azerbaijan | VI191 | 30 |
| 2013 | 38º17.399ʹN  44º71.18ʹE | Red Delicious | Salmas | West Azerbaijan | VI194 | 31 |
| 2013 | 38º17.318ʹN  44º72.401ʹE | Golden Delicious | Salmas | West Azerbaijan | VI196 | 32 |
| 2013 | 38º18.074ʹN  44º77.43ʹE | Golden Delicious | Salmas | West Azerbaijan | VI205 | 33 |
| 2013 | 38º20.448ʹN  44º80.984ʹE | Red Delicious | Salmas | West Azerbaijan | VI212 | 34 |
| 2013 | 38º19.585ʹN  44º80.555ʹE | Golden Delicious | Salmas | West Azerbaijan | VI220 | 35 |
| 2013 | 38º21.312ʹN  44º79.834ʹE | Red Delicious | Salmas | West Azerbaijan | VI222 | 36 |
| 2014 | 38º17.737ʹN  44º78.563ʹE | Red Delicious | Salmas | West Azerbaijan | VI594 | 37 |
| 2014 | 38º19.666ʹN  44º81.533ʹE | Red Delicious | Salmas | West Azerbaijan | VI604 | 38 |
| 2014 | 38º19.936ʹN  44º82.391ʹE | Red Delicious | Salmas | West Azerbaijan | VI605 | 39 |
| 2013 | 37º57.473ʹN  45º13.151ʹE | Red Delicious | Urmia | West Azerbaijan | VI182 | 40 |
| 2013 | 37º55.514ʹN  45º14.936ʹE | Red Delicious | Urmia | West Azerbaijan | VI183 | 41 |
| 2013 | 37º59.758ʹN  45º09.958ʹE | Red Delicious | Urmia | West Azerbaijan | VI184 | 42 |
| 2014 | 37º67.921ʹN  45º96.279ʹE | Red Delicious | Urmia | West Azerbaijan | VI477 | 43 |
| 2014 | 37º68.023ʹN  44º96.005ʹE | Red Delicious | Urmia | West Azerbaijan | VI478 | 44 |
| 2014 | 37º67.548ʹN  44º94.64ʹE | Red Delicious | Urmia | West Azerbaijan | VI606 | 45 |
| 2014 | 37º67.962ʹN  44º95.275ʹE | Red Delicious | Urmia | West Azerbaijan | VI608 | 46 |
| 2014 | 37º68.254ʹN  44º98.296ʹE | Red Delicious | Urmia | West Azerbaijan | VI611 | 47 |
| 2013 | 37º67.014ʹN  48º48.064ʹE | Red Delicious | Khalkhal | Ardabil | VI142 | 48 |
| 2013 | 37º67.381ʹN  48º50.897ʹE | Golden Delicious | Khalkhal | Ardabil | VI143 | 49 |
| 2013 | 37º70.302ʹN  48º44.957ʹE | Golab | Khalkhal | Ardabil | VI144 | 50 |
| 2013 | 37º70.37ʹN  48º44.305ʹE | Golab | Khalkhal | Ardabil | VI145 | 51 |
| 2013 | 37º70.397ʹN  48º44.065ʹE | Golab | Khalkhal | Ardabil | VI148 | 52 |
| 2013 | 37º70.383ʹN  48º44.271ʹE | Golab | Khalkhal | Ardabil | VI152 | 53 |
| 2014 | 38º43.280ʹN  47º65.98ʹE | Red Delicious | Meshkinshahr | Ardabil | VI525 | 54 |
| 2014 | 38º43.441ʹN  47º65.912ʹE | Red Delicious | Meshkinshahr | Ardabil | VI526 | 55 |
| 2014 | 38º42.715ʹN  47º66.392ʹE | Red Delicious | Meshkinshahr | Ardabil | VI527 | 56 |
| 2014 | 38º42.2755ʹN  47º65.654ʹE | Red Delicious | Meshkinshahr | Ardabil | VI528 | 57 |
| 2014 | 38º42.728ʹN  47º65.918ʹE | Red Delicious | Meshkinshahr | Ardabil | VI529 | 58 |
| 2014 | 38º42.581ʹN  47º66.478ʹE | Red Delicious | Meshkinshahr | Ardabil | VI530 | 59 |
| 2014 | 38º42.581ʹN  47º66.512ʹE | Red Delicious | Meshkinshahr | Ardabil | VI531 | 60 |
| 2014 | 38º42.567ʹN  47º66.787ʹE | Red Delicious | Meshkinshahr | Ardabil | VI532 | 61 |
| West population | | | | | | |
| 2014 | 35º23.52ʹN  47º1.43ʹE | Golden Delicious | Sanandaj | Kurdistan | VI521 | 62 |
| 2014 | 35º3.51ʹN  47º11.01ʹE | Golden Delicious | Sanandaj | Kurdistan | VI522 | 63 |
| 2014 | 35º22.5ʹN  47º0.07ʹE | Golden Delicious | Sanandaj | Kurdistan | VI523 | 64 |
| 2014 | 35º23.5ʹN  47º0.043ʹE | Red Delicious | Sanandaj | Kurdistan | VI584 | 65 |
| 2014 | 35º23.509ʹN  47º0.042ʹE | Red Delicious | Sanandaj | Kurdistan | VI585 | 66 |
| 2014 | 35º25.849ʹN  46º57.66ʹE | Golab | Sanandaj | Kurdistan | VI586 | 67 |
| 2014 | 35º25.848ʹN  46º57.681ʹE | Golab | Sanandaj | Kurdistan | VI588 | 68 |
| 2014 | 35º25.816ʹN  46º57.68ʹE | Golab | Sanandaj | Kurdistan | VI589 | 69 |
| 2014 | 35º25.825ʹN  46º57.686ʹE | Golab | Sanandaj | Kurdistan | VI590 | 70 |
| 2014 | 34º48.132ʹN  48º27.853ʹE | Golab | Heydareh | Hamadan | VI553 | 71 |
| 2014 | 34º48.145ʹN  48º27.87ʹE | Golab | Heydareh | Hamadan | VI554 | 72 |
| 2014 | 34º48.135ʹN  48º27.877ʹE | Golab | Heydareh | Hamadan | VI555 | 73 |
| 2014 | 34º48.13ʹN  48º27.885ʹE | Golab | Heydareh | Hamadan | VI556 | 74 |
| 2014 | 34º48.138ʹN  48º27.892ʹE | Golab | Heydareh | Hamadan | VI557 | 75 |
| 2014 | 34º48.268ʹN  48º28.202ʹE | Red Delicious | Heydareh | Hamadan | VI558 | 76 |
| 2014 | 34º48.275ʹN  48º28.215ʹE | Red Delicious | Heydareh | Hamadan | VI559 | 77 |
| 2014 | 34º48.28ʹN  48º28.221ʹE | Red Delicious | Heydareh | Hamadan | VI560 | 78 |
| 2014 | 34º48.285ʹN  48º28.23ʹE | Red Delicious | Heydareh | Hamadan | VI561 | 79 |
| 2014 | 34º48.288ʹN  48º28.235ʹE | Red Delicious | Heydareh | Hamadan | VI562 | 80 |
| 2014 | 34º48.271ʹN  48º28.214ʹE | Red Delicious | Heydareh | Hamadan | VI563 | 81 |
| 2014 | 34º48.269ʹN  48º28.169ʹE | Red Delicious | Heydareh | Hamadan | VI564 | 82 |
| 2014 | 34º48.271ʹN  48º28.227ʹE | Red Delicious | Heydareh | Hamadan | VI565 | 83 |
| 2014 | 34º48.271ʹN  48º28.213ʹE | Red Delicious | Heydareh | Hamadan | VI566 | 84 |
| 2014 | 34º48.353ʹN  48º28.661ʹE | Golab | Heydareh | Hamadan | VI567 | 85 |
| 2014 | 34º48.351ʹN  48º28.666ʹE | Golab | Heydareh | Hamadan | VI568 | 86 |
| 2014 | 34º48.355ʹN  48º28.66ʹE | Golab | Heydareh | Hamadan | VI569 | 87 |
| 2014 | 34º48.351ʹN  48º28.665ʹE | Golab | Heydareh | Hamadan | VI570 | 88 |
| 2014 | 34º48.36ʹN  48º28.67ʹE | Golab | Heydareh | Hamadan | VI571 | 89 |
| 2014 | 34º48.364ʹN  48º28.675ʹE | Golab | Heydareh | Hamadan | VI572 | 90 |
| 2014 | 34º48.358ʹN  48º28.65ʹE | Golab | Heydareh | Hamadan | VI573 | 91 |
| 2014 | 34º48.347ʹN  48º28.57ʹE | Golab | Heydareh | Hamadan | VI574 | 92 |
| 2014 | 34º48.335ʹN  48º28.525ʹE | Golab | Heydareh | Hamadan | VI575 | 93 |
| 2014 | 34º48.35ʹN  48º28.6ʹE | Golab | Heydareh | Hamadan | VI577 | 94 |
| 2014 | 34º48.344ʹN  48º28.566ʹE | Golab | Heydareh | Hamadan | VI578 | 95 |
| 2014 | 34º48.356ʹN  48º28.66ʹE | Golab | Heydareh | Hamadan | VI580 | 96 |
| 2014 | 34º48.34ʹN  48º28.6ʹE | Golab | Heydareh | Hamadan | VI581 | 97 |
| 2014 | 34º48.357ʹN  48º28.656ʹE | Golab | Heydareh | Hamadan | VI582 | 98 |
| 2014 | 34º48.349ʹN  48º28.671ʹE | Golab | Heydareh | Hamadan | VI583 | 99 |
| 2009 | 34º47.34ʹN  48º27.71ʹE | Golab | Hamadan | Hamadan | VI445 | 100 |
| 2009 | 34º47.3ʹN  48º27.61ʹE | Golab | Hamadan | Hamadan | VI446 | 101 |
| 2013 | 34º48.816ʹN  47º69.433ʹE | Golab | Sahneh | Kermanshah | VI270 | 102 |
| 2013 | 34º48.71ʹN  47º69.255ʹE | Golab | Sahneh | Kermanshah | VI271 | 103 |
| 2013 | 34º48.591ʹN  47º69.23ʹE | Golab | Sahneh | Kermanshah | VI272 | 104 |
| 2013 | 33º72.397ʹN  48º26.091ʹE | Golab | Khorramabad | Lorestan | VI258 | 105 |
| 2013 | 33º72.203ʹN  48º26.08ʹE | Golab | Khorramabad | Lorestan | VI259 | 106 |
| 2013 | 33º72.147ʹN  48º26.163ʹE | Golab | Khorramabad | Lorestan | VI260 | 107 |
| 2013 | 33º72.102ʹN  48º26.266ʹE | Golab | Khorramabad | Lorestan | VI261 | 108 |
| 2013 | 33º72.161ʹN  48º25.909ʹE | Golab | Khorramabad | Lorestan | VI262 | 109 |
| 2013 | 33º72.134ʹN  48º25.779ʹE | Golab | Khorramabad | Lorestan | VI263 | 110 |
| 2013 | 33º72.151ʹN  48º25.698ʹE | Golab | Khorramabad | Lorestan | VI264 | 111 |
| Central population | | | | | | |
| 2013 | 35º92.905ʹN  51º07.984ʹE | Golab | Chalus road, Morud | Alborz | VI1 | 112 |
| 2013 | 35º92.337ʹN  51º08.116ʹE | Moruei | Chalus road, Morud | Alborz | VI2 | 113 |
| 2013 | 35º92.89ʹN  51º07.87ʹE | Golab | Chalus road, Morud | Alborz | VI3 | 114 |
| 2013 | 35º92.145ʹN  51º07.976ʹE | Shafiabadi | Chalus road, Morud | Alborz | VI8 | 115 |
| 2013 | 35º92.188ʹN  51º08.01ʹE | Moruei | Chalus road, Morud | Alborz | VI13 | 116 |
| 2013 | 35º92.059ʹN  51º08.397ʹE | Golab | Chalus road, Arangeh | Alborz | VI25 | 117 |
| 2013 | 35º92.031ʹN  51º09.015ʹE | Golab | Chalus road, Arangeh | Alborz | VI28 | 118 |
| 2013 | 35º90.868ʹN  51º12.461ʹE | Rasmi | Chalus road, Charan | Alborz | VI32 | 119 |
| 2013 | 35º91.697ʹN  51º10.938ʹE | Golab | Chalus road, Sarziarat | Alborz | VI43 | 120 |
| 2013 | 35º91.614ʹN  51º10.869ʹE | Golab | Chalus road, Sarziarat | Alborz | VI46 | 121 |
| 2013 | 35º55.088ʹN  51º7.118ʹE | Golab | Chalus road, Jey | Alborz | VI47 | 122 |
| 2014 | 36º2.404ʹN  51º11.924ʹE | Golab | Chalus road, Asara | Alborz | VI448 | 123 |
| 2013 | 35º89.098ʹN  50º71.662ʹE | Golden Delicious | Hashtgerd | Alborz | VI59 | 124 |
| 2013 | 35º97.327ʹN  50º61.826ʹE | Golab | Nazarabad | Alborz | VI246 | 125 |
| 2013 | 35º97.285ʹN  50º61.774ʹE | Golab | Nazarabad | Alborz | VI249 | 126 |
| 2013 | 35º97.41ʹN  50º61.963ʹE | Golab | Nazarabad | Alborz | VI256 | 127 |
| 2013 | 35º97.313ʹN  50º61.929ʹE | Golab | Nazarabad | Alborz | VI257 | 128 |
| 2013 | 36º9.25ʹN  50º43.91ʹE | Golab | Taleghan | Alborz | VI251 | 129 |
| 2014 | 36º10.256ʹN  50º44.919ʹE | Red Delicious | Taleghan | Alborz | VI330 | 130 |
| 2014 | 36º9.773ʹN  50º41.769ʹE | Golab | Taleghan | Alborz | VI336 | 131 |
| 2014 | 36º9.919ʹN  50º43.134ʹE | Golab | Taleghan | Alborz | VI339 | 132 |
| 2014 | 36º10.257ʹN  50º44.92ʹE | Golden Delicious | Taleghan | Alborz | VI342 | 133 |
| 2014 | 36º10.244ʹN  50º44.91ʹE | Golden Delicious | Taleghan | Alborz | VI345 | 134 |
| 2014 | 36º9.894ʹN  50º43.002ʹE | Golab | Taleghan | Alborz | VI348 | 135 |
| 2014 | 35º57.159ʹN  50º55.806ʹE | Golab | Baraghan | Alborz | VI540 | 136 |
| 2014 | 35º57.172ʹN  50º55.763ʹE | Golab | Baraghan | Alborz | VI543 | 137 |
| 2014 | 35º57.167ʹN  50º55.771ʹE | Golab | Baraghan | Alborz | VI544 | 138 |
| 2014 | 35º57.169ʹN  50º55.766ʹE | Golab | Baraghan | Alborz | VI545 | 139 |
| 2013 | 36º70.963ʹN  48º37.992ʹE | Golab | Zanjan | Zanjan | VI228 | 140 |
| 2013 | 36º70.797ʹN  48º38.988ʹE | Golab | Zanjan | Zanjan | VI230 | 141 |
| 2013 | 36º70.027ʹN  48º38.713ʹE | Golab | Zanjan | Zanjan | VI231 | 142 |
| 2013 | 36º68.595ʹN  48º42.421ʹE | Golab | Zanjan | Zanjan | VI236 | 143 |
| 2013 | 36º67.549ʹN  48º43.554ʹE | Golab | Zanjan | Zanjan | VI238 | 144 |
| 2013 | 36º22.564ʹN  49º18.235ʹE | Golab | Khorramdarreh | Zanjan | VI241 | 145 |
| 2013 | 36º23.311ʹN  49º17.523ʹE | Golab | Khorramdarreh | Zanjan | VI242 | 146 |
| 2013 | 36º63.693ʹN  48º54.265ʹE | Golab | Zanjan | Zanjan | VI267 | 147 |
| 2013 | 36º63.307ʹN  48º58.351ʹE | Golab | Zanjan | Zanjan | VI268 | 148 |
| 2013 | 36º65.897ʹN  48º56.154ʹE | Golab | Zanjan | Zanjan | VI269 | 149 |
| 2014 | 36º47.499ʹN  48º13.371ʹE | Red Delicious | Zanjan | Zanjan | VI591 | 150 |
| 2014 | 36º47.572ʹN  48º15.471ʹE | Golab | Zanjan | Zanjan | VI592 | 151 |
| 2014 | 36º12.610ʹN  50º0.278ʹE | Golab | Qadimabad | Qazvin | VI316 | 152 |
| 2014 | 36º12.618ʹN  50º0.284ʹE | Golab | Qadimabad | Qazvin | VI317 | 153 |
| 2014 | 36º12.621ʹN  50º0.311ʹE | Golab | Qadimabad | Qazvin | VI318 | 154 |
| 2014 | 36º12.732ʹN  49º58.803ʹE | Golab | Qadimabad | Qazvin | VI321 | 155 |
| 2014 | 36º12.74ʹN  49º58.81ʹE | Golab | Qadimabad | Qazvin | VI323 | 156 |
| 2014 | 36º12.619ʹN  50º0.298ʹE | Golab | Qadimabad | Qazvin | VI324 | 157 |
| 2014 | 35º66.718ʹN  51º04.556ʹE | Golab | Shahriar | Tehran | VI307 | 158 |
| 2014 | 35º5.114ʹN  49º49.273ʹE | Golab | Saveh | Markazi | VI280 | 159 |
| 2014 | 35º5.124ʹN  49º49.274ʹE | Golab | Saveh | Markazi | VI281 | 160 |
| 2014 | 35º5.123ʹN  49º49.277ʹE | Golab | Saveh | Markazi | VI282 | 161 |
| 2014 | 35º5.123ʹN  49º49.276ʹE | Golab | Saveh | Markazi | VI283 | 162 |
| 2014 | 35º5.111ʹN  49º49.276ʹE | Golab | Saveh | Markazi | VI284 | 163 |
| 2014 | 35º5.119ʹN  49º49.273ʹE | Golab | Saveh | Markazi | VI286 | 164 |
| 2013 | 31º39.726ʹN  51º56.161ʹE | Golab | Semirom | Isfahan | VI85 | 165 |
| North population | | | | | | |
| 2014 | 37º35.671ʹN  49º1.736ʹE | *Malus orientalis* | Paresar | Guilan | VI291 | 166 |
| 2014 | 37º35.679ʹN  49º1.739ʹE | *Malus orientalis* | Paresar | Guilan | VI292 | 167 |
| 2014 | 37º8.23ʹN  49º39.9ʹE | *Malus orientalis* | Saravan Park | Guilan | VI293 | 168 |
| 2014 | 37º8.227ʹN  49º39.915ʹE | *Malus orientalis* | Saravan Park | Guilan | VI294 | 169 |
| 2014 | 37º8.198ʹN  49º39.921ʹE | *Malus orientalis* | Saravan Park | Guilan | VI295 | 170 |
| 2014 | 37º12.195ʹN  49º59.467ʹE | *Malus orientalis* | Lahijan | Guilan | VI296 | 171 |
| 2014 | 37º9.881ʹN  49º58.023ʹE | Golab | Lahijan | Guilan | VI300 | 172 |
| 2014 | 37º32.158ʹN  48º57.085ʹE | Golab | Rezvanshahr-Punel | Guilan | VI533 | 173 |
| 2014 | 37º32.196ʹN  48º57.165ʹE | Golab | Rezvanshahr-Punel | Guilan | VI534 | 174 |
| 2014 | 37º32.164ʹN  48º57.032ʹE | Golab | Rezvanshahr-Punel | Guilan | VI535 | 175 |
| 2014 | 37º32.153ʹN  48º57.058ʹE | Golab | Rezvanshahr-Punel | Guilan | VI536 | 176 |
| 2014 | 37º30.158ʹN  48º5.467ʹE | Golab | Rezvanshahr-Punel | Guilan | VI537 | 177 |
| 2014 | 37º30.162ʹN  48º5.5ʹE | Golab | Rezvanshahr-Punel | Guilan | VI538 | 178 |
| 2014 | 37º10.177ʹN  49º16.591ʹE | Golab | Fuman | Guilan | VI539 | 179 |
| 2014 | 36º38.94ʹN  51º25.934ʹE | Golab | Chalus | Mazandaran | VI304 | 180 |
| 2014 | 36º38.85ʹN  51º25.9ʹE | Golab | Chalus | Mazandaran | VI305 | 181 |
| 2014 | 36º12.006ʹN  51º48.623ʹE | Red Delicious | Nour, Baladeh | Mazandaran | VI355 | 182 |
| 2014 | 36º12.019ʹN  51º48.607ʹE | Golab | Nour, Baladeh | Mazandaran | VI356 | 183 |
| 2014 | 36º12.018ʹN  52º.06ʹE | Golden Delicious | Nour, Baladeh,Takur | Mazandaran | VI357 | 184 |
| 2014 | 36º12.022ʹN  52º1.092ʹE | Red Delicious | Nour, Baladeh, Takur | Mazandaran | VI358 | 185 |
| 2014 | 36º12.127ʹN  51º55.342ʹE | Golden Delicious | Nour, Valashit | Mazandaran | VI361 | 186 |
| 2014 | 36º26.68ʹN  51º54.459ʹE | Golab | Nour, Galandrud | Mazandaran | VI362 | 187 |
| 2014 | 36º26.689ʹN  51º54.473ʹE | Karaj apple | Nour, Galandrud | Mazandaran | VI363 | 188 |
| 2014 | 36º12.119ʹN  51º55.348ʹE | Golden Delicious | Nour, Valashit | Mazandaran | VI364 | 189 |
| 2014 | 36º12.065ʹN  51º51.916ʹE | Red Delicious | Nour, Davilat | Mazandaran | VI367 | 190 |
| 2014 | 36º12.031ʹN  51º48.604ʹE | Red Delicious | Nour, Baladeh | Mazandaran | VI370 | 191 |
| 2014 | 36º12.038ʹN  51º51.892ʹE | Golden Delicious | Nour, Baladeh | Mazandaran | VI371 | 192 |
| 2014 | 36º12.031ʹN  51º48.601ʹE | Golab | Nour, Davilat | Mazandaran | VI372 | 193 |
| 2014 | 36º12.033ʹN  51º51.877ʹE | Golden Delicious | Nour, Davilat | Mazandaran | VI373 | 194 |
| 2014 | 36º12.04ʹN  51º51.875ʹE | Golden Delicious | Nour, Davilat | Mazandaran | VI375 | 195 |
| 2014 | 36º12.033ʹN  51º51.867ʹE | Golden Delicious | Nour, Davilat | Mazandaran | VI376 | 196 |
| 2014 | 36º11.349ʹN  52º29.553ʹE | *Malus Orientalis* | Amol, Sangchal | Mazandaran | VI407 | 197 |
| 2014 | 36º11.571ʹN  52º29.984ʹE | Sibe Sorkh | Amol, Sangchal | Mazandaran | VI410 | 198 |
| 2014 | 36º11.772ʹN  52º28.477ʹE | Sibe Sang | Amol, Chameban | Mazandaran | VI411 | 199 |
| 2014 | 36º11.638ʹN  52º28.072ʹE | Sibe Sorkh | Amol, Kangrajkola | Mazandaran | VI412 | 200 |
| 2014 | 36º11.571ʹN  52º28.072ʹE | Golab | Amol, Kangrajkola | Mazandaran | VI413 | 201 |
| 2014 | 36º12.653ʹN  52º25.615ʹE | Golab | Amol, Chelav | Mazandaran | VI416 | 202 |
| 2014 | 36º29.31ʹN  51º20.198ʹE | Golab | Noshahr, Kojur | Mazandaran | VI420 | 203 |
| 2014 | 36º29.311ʹN  51º20.195ʹE | Ghandak | Noshahr, Kojur | Mazandaran | VI422 | 204 |
| 2014 | 36º26.573ʹN  51º23.173ʹE | *Malus orientalis* | Noshahr, Neyres forest | Mazandaran | VI424 | 205 |
| 2014 | 36º26.571ʹN  51º23.175ʹE | *Malus orientalis* | Noshahr, Neyres forest | Mazandaran | VI425 | 206 |
| 2014 | 36º26.566ʹN  51º23.178ʹE | *Malus orientalis* | Noshahr, Neyres forest | Mazandaran | VI426 | 207 |
| 2014 | 36º26.407ʹN  51º23.217ʹE | *Malus orientalis* | Noshahr, Neyres forest | Mazandaran | VI429 | 208 |
| 2014 | 36º26.575ʹN  51º23.171ʹE | *Malus orientalis* | Noshahr, Neyres forest | Mazandaran | VI430 | 209 |
| 2014 | 36º26.678ʹN  51º22.956ʹE | *Malus orientalis* | Noshahr, Neyres forest | Mazandaran | VI431 | 210 |
| 2014 | 36º26.722ʹN  51º23.056ʹE | *Malus orientalis* | Noshahr, Neyres forest | Mazandaran | VI433 | 211 |
| 2014 | 36º66.251ʹN  53º06.978ʹE | Golab | Sari | Mazandaran | VI480 | 212 |
| 2014 | 36º66.246ʹN  53º06.979ʹE | Golab | Sari | Mazandaran | VI482 | 213 |
| 2014 | 36º23.637ʹN  53º53.576ʹE | Golab | Kiasar | Mazandaran | VI483 | 214 |
| 2014 | 36º23.53ʹN  53º53.597ʹE | Golab | Kiasar | Mazandaran | VI485 | 215 |
| 2014 | 36º38.073ʹN  52º23.773ʹE | Golab | Kordkuy | Golestan | VI378 | 216 |
| 2014 | 36º38.31ʹN  54º5.639ʹE | Golab | Kordkuy | Golestan | VI379 | 217 |
| 2014 | 36º38.319ʹN  54º5.646ʹE | Golab | Kordkuy | Golestan | VI380 | 218 |
| 2014 | 36º38.473ʹN  54º5.726ʹE | Golab | Kordkuy | Golestan | VI381 | 219 |
| 2014 | 37º18.035ʹN  55º39.52ʹE | *Malus orientalis* | Gorgan-Loveh forest | Golestan | VI386 | 220 |
| 2014 | 37º18.013ʹN  55º39.556ʹE | *Malus orientalis* | Gorgan-Loveh forest | Golestan | VI387 | 221 |
| 2014 | 37º18.033ʹN  55º39.507ʹE | *Malus orientalis* | Gorgan-Loveh forest | Golestan | VI389 | 222 |
| 2014 | 37º18.029ʹN  55º39.522ʹE | *Malus orientalis* | Gorgan-Loveh forest | Golestan | VI390 | 223 |
| 2014 | 37º18.005ʹN  55º39.576ʹE | *Malus orientalis* | Gorgan-Loveh forest | Golestan | VI391 | 224 |
| 2014 | 37º18.004ʹN  55º39.57ʹE | *Malus orientalis* | Gorgan-Loveh forest | Golestan | VI392 | 225 |
| 2014 | 37º18.014ʹN  55º39.577ʹE | *Malus orientalis* | Gorgan-Loveh forest | Golestan | VI393 | 226 |
| 2014 | 37º17.987ʹN  55º39.519ʹE | *Malus orientalis* | Gorgan-Loveh forest | Golestan | VI395 | 227 |
| Northeast population | | | | | | |
| 2014 | 41º44.208ʹN  47º54.86ʹE | Golden Delicious | Bojnord | North Khorasan | VI450 | 228 |
| 2014 | 41º44.136ʹN  47º54.79ʹE | Golden Delicious | Bojnord | North Khorasan | VI451 | 229 |
| 2014 | 41º44.165ʹN  47º54.82ʹE | Golden Delicious | Bojnord | North Khorasan | VI452 | 230 |
| 2014 | 41º27.836ʹN  53º26.76ʹE | Golden Delicious | Bojnord | North Khorasan | VI453 | 231 |
| 2014 | 41º38.307ʹN  52º96.17ʹE | Red Delicious | Bojnord | North Khorasan | VI454 | 232 |
| 2014 | 41º38.244ʹN  52º96.41ʹE | Golden Delicious | Bojnord | North Khorasan | VI455 | 233 |
| 2014 | 41º27.930ʹN  53º27.12ʹE | Golab | Bojnord | North Khorasan | VI456 | 234 |
| 2014 | 41º46.007ʹN  47º65.18ʹE | Golab | Bojnord | North Khorasan | VI457 | 235 |
| 2014 | 41º45.987ʹN  47º65.06ʹE | Golab | Bojnord | North Khorasan | VI458* | 236 |
| 2014 | 41º44.409ʹN  47º55.68ʹE | Golab | Bojnord | North Khorasan | VI459 | 237 |
| 2014 | 41º44.310ʹN  47º56.12ʹE | Golab | Bojnord | North Khorasan | VI460 | 238 |
| 2014 | 41º44.322ʹN  47º56.05ʹE | Golab | Bojnord | North Khorasan | VI461 | 239 |
| 2014 | 41º44.335ʹN  47º56.13ʹE | Golab | Bojnord | North Khorasan | VI462 | 240 |
| 2014 | 41º44.394ʹN  47º55.26ʹE | Golab | Bojnord | North Khorasan | VI463 | 241 |
| 2014 | 41º38.371ʹN  52º96.67ʹE | Golab | Bojnord | North Khorasan | VI464 | 242 |
| 2014 | 41º27.938ʹN  53º26.93ʹE | Golab | Bojnord | North Khorasan | VI465 | 243 |
| 2014 | 41º27.3811ʹN  53º27.02ʹE | Golden Delicious | Bojnord | North Khorasan | VI466 | 244 |
| 2014 | 41º38.32ʹN  52º96.28ʹE | Red Delicious | Bojnord | North Khorasan | VI467 | 245 |
| 2014 | 37º42.571ʹN  57º31.159ʹE | Golden Delicious | Bojnord | North Khorasan | VI468 | 246 |
| 2014 | 37º42.65ʹN  57º31.169ʹE | Golden Delicious | Bojnord | North Khorasan | VI469 | 247 |
| 2014 | 37º42.589ʹN  57º31.2ʹE | Golden Delicious | Bojnord | North Khorasan | VI470 | 248 |
| 2014 | 37º41.415ʹN  57º33.851ʹE | Golab | Bojnord | North Khorasan | VI471 | 249 |
| 2014 | 37º42.455ʹN  57º34.342ʹE | Golab | Bojnord | North Khorasan | VI472 | 250 |
| 2014 | 37º42.937ʹN  57º34.124ʹE | Golden Delicious | Bojnord | North Khorasan | VI473 | 251 |
| 2014 | 37º43.178ʹN  57º34.695ʹE | Golab | Bojnord | North Khorasan | VI486 | 252 |
| 2014 | 37º42.543ʹN  57º31.195ʹE | Golab | Bojnord | North Khorasan | VI487 | 253 |
| 2014 | 37º44.092ʹN  57º32.775ʹE | Golab | Bojnord | North Khorasan | VI488 | 254 |
| 2014 | 37º43.706ʹN  57º32.702ʹE | Golab | Bojnord | North Khorasan | VI489 | 255 |
| 2014 | 37º44.131ʹN  57º33.104ʹE | Golab | Bojnord | North Khorasan | VI490 | 256 |
| 2014 | 37º4.7ʹN  58º38.441ʹE | Red Delicious | Quchan | Razavi Khorasan | VI491 | 257 |
| 2014 | 37º4.67ʹN  58º38.431ʹE | Abbasi | Quchan | Razavi Khorasan | VI492 | 258 |
| 2014 | 37º4.682ʹN  58º38.436ʹE | Red Delicious | Quchan | Razavi Khorasan | VI493 | 259 |
| 2014 | 37º4.714ʹN  58º38.445ʹE | Red Delicious | Quchan | Razavi Khorasan | VI495 | 260 |
| 2014 | 37º2.865ʹN  58º48.923ʹE | Golden Delicious | Quchan | Razavi Khorasan | VI497 | 261 |
| 2014 | 37º2.86ʹN  58º48.9ʹE | Red Delicious | Quchan | Razavi Khorasan | VI498* | 262 |
| 2014 | 37º2.95ʹN  58º48.866ʹE | Red Delicious | Quchan | Razavi Khorasan | VI499 | 263 |
| 2014 | 37º2.876ʹN  58º48.923ʹE | Red Delicious | Quchan | Razavi Khorasan | VI501 | 264 |
| 2014 | 37º2.883ʹN  58º48.925ʹE | Red Delicious | Quchan | Razavi Khorasan | VI502 | 265 |
| 2014 | 37º2.942ʹN  58º48.892ʹE | Golden Delicious | Quchan | Razavi Khorasan | VI505 | 266 |
| 2014 | 37º2.958ʹN  58º48.849ʹE | Golden Delicious | Quchan | Razavi Khorasan | VI506 | 267 |
| 2014 | 37º3.689ʹN  58º47.607ʹE | Red Delicious | Quchan | Razavi Khorasan | VI507 | 268 |
| 2014 | 37º3.663ʹN  58º47.61ʹE | Red Delicious | Quchan | Razavi Khorasan | VI508 | 269 |
| 2014 | 37º2.962ʹN  58º48.852ʹE | Red Delicious | Quchan | Razavi Khorasan | VI509 | 270 |
| 2014 | 37º3.735ʹN  58º47.668ʹE | Red Delicious | Quchan | Razavi Khorasan | VI510 | 271 |
| 2014 | 37º3.715ʹN  58º47.647ʹE | Red Delicious | Quchan | Razavi Khorasan | VI511 | 272 |
| 2014 | 37º3.689ʹN  58º47.632ʹE | Red Delicious | Quchan | Razavi Khorasan | VI512 | 273 |
| 2014 | 37º3.684ʹN  58º47.617ʹE | Red Delicious | Quchan | Razavi Khorasan | VI513 | 274 |
| 2014 | 37º3.637ʹN  58º47.609ʹE | Golden Delicious | Quchan | Razavi Khorasan | VI514 | 275 |
| 2014 | 37º3.637ʹN  58º47.607ʹE | Golden Delicious | Quchan | Razavi Khorasan | VI515 | 276 |
| 2014 | 37º2.949ʹN  58º48.854ʹE | Golab | Quchan | Razavi Khorasan | VI516 | 277 |
| 2014 | 37º2.949ʹN  58º48.869ʹE | Red Delicious | Quchan | Razavi Khorasan | VI517 | 278 |
| 2014 | 37º4.676ʹN  58º38.431ʹE | Abbasi | Quchan | Razavi Khorasan | VI518 | 279 |
| 2014 | 37º2.943ʹN  58º48.879ʹE | Red Delicious | Quchan | Razavi Khorasan | VI519 | 280 |

*Isolates in red font did not amplify with the SSR markers and hence were not included in further genetic analysis.
